# The potential convergence of NLRP3 inflammasome, potassium, and dopamine mechanisms in Parkinson’s disease

**DOI:** 10.1038/s41531-022-00293-z

**Published:** 2022-03-24

**Authors:** Adrianne F. Pike, Ildikò Szabò, Robert Veerhuis, Luigi Bubacco

**Affiliations:** 1 Amsterdam UMC, Vrije Universiteit Amsterdam, Neurochemistry Laboratory, Department of Clinical Chemistry, Amsterdam Neuroscience, Amsterdam, the Netherlands; 2 Department of Biology, University of Padua, Padua, Italy; 3 CNR Institute of Neuroscience, Padua, Italy; 4 Amsterdam UMC, Vrije Universiteit Amsterdam, Department of Psychiatry, Amsterdam Neuroscience, Amsterdam, the Netherlands

**Keywords:** Neuroimmunology, Parkinson's disease

## Abstract

The pathology of Parkinson’s disease (PD) is characterized by α-synuclein aggregation, microglia-mediated neuroinflammation, and dopaminergic neurodegeneration in the substantia nigra with collateral striatal dopamine signaling deficiency. Microglial NLRP3 inflammasome activation has been linked independently to each of these facets of PD pathology. The voltage-gated potassium channel Kv1.3, upregulated in microglia by α-synuclein and facilitating potassium efflux, has also been identified as a modulator of neuroinflammation and neurodegeneration in models of PD. Evidence increasingly suggests that microglial Kv1.3 is mechanistically coupled with NLRP3 inflammasome activation, which is contingent on potassium efflux. Potassium conductance also influences dopamine release from midbrain dopaminergic neurons. Dopamine, in turn, has been shown to inhibit NLRP3 inflammasome activation in microglia. In this review, we provide a literature framework for a hypothesis in which Kv1.3 activity-induced NLRP3 inflammasome activation, evoked by stimuli such as α-synuclein, could lead to microglia utilizing dopamine from adjacent dopaminergic neurons to counteract this process and fend off an activated state. If this is the case, a sufficient dopamine supply would ensure that microglia remain under control, but as dopamine is gradually siphoned from the neurons by microglial demand, NLRP3 inflammasome activation and Kv1.3 activity would progressively intensify to promote each of the three major facets of PD pathology: α-synuclein aggregation, microglia-mediated neuroinflammation, and dopaminergic neurodegeneration. Risk factors overlapping to varying degrees to render brain regions susceptible to such a mechanism would include a high density of microglia, an initially sufficient supply of dopamine, and poor insulation of the dopaminergic neurons by myelin.

## Background: Parkinson’s disease pathology and the microglial NLRP3 inflammasome

Parkinson’s disease (PD) is the most prevalent movement disorder and the second most prevalent neurodegenerative disease, only Alzheimer’s disease being more common^1–4^. Though familial PD, involving mutations in genes for disease-relevant proteins including *LRRK2*, *PARK7, PINK1, DJ1, PRKN*, and *SNCA*, accounts for a small proportion of cases, approximately 90% of PD cases are sporadic, with no readily identified catalyst for pathology^1,2,5–9^. External factors such as elevated levels of heavy metals like manganese or copper^10,11^ or exposure to pesticides like the electron transport chain Complex I inhibitor rotenone or paraquat^12^ have been implicated in the etiology of PD^13^, presumably exerting their effects by interfering with mitochondrial function and generating increased levels of reactive oxygen species, but age is the most important risk factor for the disease, which occurs in about 1–2% of the over-60 population^1,14–17^. As the population ages, more time and cost will be invested into treatments for PD patients, and the pressing need for improved interventions and etiological insight into both the familial and sporadic forms of the disease is evident^18^.

The pathology of PD typically displays three cardinal features. One is the accumulation of the protein α-synuclein, leading to the formation of intraneuronal inclusions known as Lewy bodies (LBs) and Lewy neurites (LNs) in areas of neurodegeneration^1,19–22^. Another key characteristic of PD is neuroinflammation, hallmarked by the presence of activated microglia which assemble around degenerating dopaminergic neurons^23,24^. This dopaminergic neurodegeneration represents the third major facet of PD pathology, taking place most prominently in the *substantia nigra pars compacta* (SNpc) region of the midbrain and leading to impaired striatal dopamine signaling^25–28^. The ramifications of inadequate dopamine signaling in the basal ganglia are clear in PD, as motor symptoms such as akinesia and tremor develop as a result^29^.

## α-Synuclein

α-Synuclein (α-syn) is an ~14 kDa protein with its most prevalent expression in brain tissue. It is primarily expressed by neurons, where it is localized to the presynaptic terminal and the nucleus (hence its name synuclein^22,30^), and it is thought to be involved under baseline conditions in the transport of intracellular vesicles^31–33^. α-Syn contains seven repeats of a synuclein family-specific amino acid sequence containing the consensus residues KTKEGV^22,34^. These repeats have been shown to be involved in normal tetramerization of the α-syn peptide, and alterations in the consensus sequence are associated with increased neurotoxicity^34^. α-Syn is an intrinsically disordered protein^35^ with a propensity to misfold and aggregate into cross-β-sheet-rich fibrils and oligomers^9,15,22,36^, especially upon irreversible truncation at its C-terminal^6,37^. The C-terminal truncated α-syn is more likely to aggregate than full-length α-syn^32^, and it is pervasive in PD-associated LBs but is typically not found in soluble form^37^. While PD is the most frequently encountered synucleinopathy, α-syn aggregation pathology also occurs in other diseases such as dementia with Lewy bodies (DLB) and multiple system atrophy (MSA)^22,32,38–42^.

Soluble α-syn is known to be secreted from neurons into the extracellular space, and microglia have been suggested to be the primary cell type responsible for its clearance^43^. α-Syn has been shown to activate microglia after phagocytosis, contributing specifically to dopaminergic neurodegeneration in a murine primary neural-glia coculture model of parkinsonism^21^. In fact, α-syn has been reported to activate microglia tenfold more than Alzheimer’s disease-associated Aβ^21^. α-Syn-activated microglia were shown to be critical for dopaminergic neurodegeneration in a rat primary cell PD model, and phagocytosis of the α-syn by microglia was imperative to this process^21^.

PD-associated neuroinflammation is typified by the presence of activated microglia, the resident immune cells of the brain, which assemble around degenerating neurons^23,24,44^. Microglia-mediated cross-talk with neurons plays a major role in the pathology of PD^45^. The SNpc is, notably, the brain region in which the density of microglia is the highest^21,23,24,46–48^. A major role in the perpetuation of PD-associated neuroinflammation is played by the pro-inflammatory cytokine interleukin-1β (IL-1β), which is secreted in the central nervous system (CNS) primarily by microglia acting as immune effectors^37,49–51^. While elevated levels of other cytokines such as TNF-α and IL-6 are also associated with microglia-mediated neuroinflammation in neurodegenerative diseases including PD, IL-1β is increased in the brain and CSF of both PD and AD patients^51^ and in the dopaminergic striatal tissue and cerebrospinal fluid of PD patients^51–54^. These observations suggest a potential mechanistic link between IL-1β production, particularly by microglia, and PD pathology in vivo.

## The NLRP3 inflammasome

The canonical mechanism for IL-1β release from microglia and peripheral myeloid cells involves activation of the NLRP3 inflammasome^49,55,56^, which is a multimolecular scaffold whose primary function is to amplify and broadcast pro-inflammatory signals from one cell to another by driving the secretion of both IL-1β and, to a lesser extent, IL-18^57–59^. Highly expressed by microglia, the three traditionally accepted component proteins of the NLRP3 inflammasome are: (1) the intracellular pattern recognition receptor *NACHT domain-, leucine-rich repeat (LRR)-, and pyrin domain-containing protein 3* (NLRP3), which oligomerizes upon efflux of potassium (K^+^) from the cell to recruit (2) the small adapter protein known as *apoptosis-associated speck-like protein containing a caspase activation and recruitment domain* (CARD), or ASC, which further oligomerizes to recruit (3) the *cysteine-aspartate protease-1* (caspase-1)^20,56,57,60^. Caspase-1’s recruitment to the inflammasome scaffold results in an increase of its local concentration, leading to its proximity-induced auto-activation, and caspase-1 is then released from the complex in its active p33/p10 form both to process IL-1β for secretion and downstream signaling and to intrinsically cue the cessation of inflammasome activation^57,61^.

Because IL-1b is such a potent inflammatory signal, its production by the NLRP3 inflammasome is regulated on multiple levels^62^. Upon their initial expression, IL-1β and IL-18 are both “leaderless” cytokines, lacking the hydrophobic targeting sequence for exocytosis that other cytokines like IL-6 possess and thus requiring further processing by cleavage for activation and secretion from the cell^63^. IL-1β is initially produced in its inactive zymogen form (pro-IL-1β), and NLRP3 inflammasome activation must take place to facilitate IL-1β maturation via caspase-mediated cleavage. NLRP3 inflammasome activation requires two separate, sequential signals for scaffold assembly and the subsequent cleavage of pro- to active IL-1β by caspase-1. The first signal, known as priming, typically involves ligand binding to cell surface receptors such as IL-1 receptors, tissue necrosis factor (TNF) receptors, or Toll-like receptors (TLRs), and thus, the molecular natures of priming signals are diverse. Priming precipitates the translocation from the cytosol to the nucleus of the transcription factor NF-κB, which, among other functions, elicits the upregulation of both NLRP3 and pro-IL-1β^59^. The second signal, known as activation, also varies mechanistically, but increasing evidence suggests that differential activation signals converge on the efflux of potassium ions (K^+^) from the cell as a consensus signal to trigger the oligomerization of NLRP3 and the ensuing recruitment of ASC and caspase-1 to form the rest of the inflammasome scaffold to process IL-1β^64–66^.

α-Syn aggregates have been shown in vitro to act as an NLRP3 inflammasome inducer in various human cell types: primary monocytes^7^, in THP-1-derived, macrophage-like cells^67,68^, and in primary microglia^67^, all leading to the processing and secretion of mature IL-1β into the culture supernatant. Evidence of in vivo activation of the NLRP3 inflammasome has been detected in association with α-syn in the affected brain tissue of PD patients; notably, caspase-1 is present alongside α-syn in Lewy bodies with a spatially ordered distribution^37^. This spatially ordered distribution may be a result of the caspase’s binding pattern on the inflammasome scaffold and indicative of the inflammasome’s role in Lewy body formation. It has been suggested that NLRP3 inflammasome initiation specifically by α-syn plays a role in the emergence and facilitation of progressive PD pathology^7,68^.

A biochemical relationship between canonically activated caspase-1 and α-syn has been demonstrated in which the active caspase cleaves α-syn at Asp121 to a truncated form (α-syn 1–121) that is more prone to aggregation and is present in Lewy bodies in vivo^37^. In the same study, caspase-1 was detected in association with α-syn in Lewy bodies in brain tissue from PD patients, leading the authors to hypothesize that α-syn aggregation and Lewy body formation in neurons might be promoted over time by caspase-1 activity. This relationship between caspase-1 activity and α-syn may account for our observation that caspase-1, though it was recruited to the inflammasome scaffold and thus presumably activated, was not necessary for IL-1β processing by primary human microglia in response to α-syn activation^67^. If caspase-1 is diverted to α-syn processing in α-syn-activated human microglia, the task of IL-1β processing in response to NLRP3 inflammasome activation would then likely fall to other caspases or proteases.

## Alternative caspase activation mechanisms

Indeed, apart from the caspase-1-dependent canonical inflammasome pathway, an alternative mechanism for NLRP3 inflammasome activation has been described for monocytes and monocyte-derived macrophages of humans, nonhuman primates, and mice. Known as noncanonical inflammasome activation, this mechanism occurs in response to the intracellular uptake of bacterial lipopolysaccharide (LPS) after an exposure time of 16 h or more^69–73^. The internalized LPS binds directly to the CARDs of caspases other than caspase-1, which then serve as cytosolic rather than surface LPS receptors to induce IL-1β secretion^74^. The caspases responsible for this process belong to the caspase-1 subfamily: caspases-4 and -5 in humans and nonhuman primates^69,70,72,73,75^ and caspase-11, the murine homolog of caspases-4 and -5, in mice^74,76–78^. Activation of noncanonical inflammasomes, particularly by triggers other than LPS, has not yet been fully characterized, and which caspases in particular are involved in NLRP3 inflammasome activation in primary human microglia, especially in neurodegenerative disease-related conditions, remains uncertain. Unpublished work from our group suggests that caspases-4 and -5, along with caspase-8, play a role in α-syn-mediated NLRP3 inflammasome activation in primary human microglia (Pike et al., manuscript in preparation).

Interestingly, both caspases-1 and -8 regulate the protein parkin^79^. Parkin, encoded by the genes *PARK2* and *PARK6*, is an E3 ligase that ubiquitinates NLRP3 among other proteins^80^. Loss-of-function mutations of parkin are associated with familial PD, and both microglia from *PARK2* knockout in vivo models and peripheral macrophages from patients with *PARK2* mutations displayed enhanced IL-1β and IL-18 output^80^. These observations link inflammasome regulation and specific caspases to inherited as well as sporadic forms of PD.

While another of the major facets of PD, dopaminergic neurodegeneration, has long been known to require microglia in several in vivo and in vitro models^21,45,81,82^, the focus on the mechanistic role of microglia in this process has narrowed further to include NLRP3 inflammasome activation in particular. Inhibition of NLRP3 by MCC950 (CRID3) or knockdown of NLRP3 or caspase-1 expression spares dopaminergic neurons from microglia-mediated degeneration^14,83–85^. The specific mechanistic nature of the dependence of dopaminergic neurodegeneration on the inflammasome is not yet known, though the links between the NLRP3 inflammasome and PD pathology are becoming increasingly clear. NLRP3 inflammasome components have been observed in the pathological brain tissue of in vivo parkinsonism mouse models as well as in human PD brain, produced by activated microglia in particular^14^. NLRP3 and ASC have been shown to be upregulated in substantia nigra microglia of PD patients and have also been detected in the striatal microglia of mouse models of parkinsonism^14^, implicating the NLRP3 inflammasome as a key agent in PD pathology. Degeneration is largely relegated to the dopaminergic neurons of the SNpc, and the mechanistic reason for their vulnerability relative to the dopaminergic neurons of the neighboring ventral tegmental area (VTA) is a subject of ongoing investigation^86,87^.

## Microglia and PD pathology: potassium efflux

K^+^ efflux is widely accepted as a consensus step for the assembly of the NLRP3 inflammasome scaffold^64–66,88–92^. This dependence has been attributed to the action of the protein NEK7 (never-in-mitosis/NIMA-related kinase 7), which is recruited to the NLRP3 inflammasome complex and is crucial for its activation downstream of K^+^ efflux^66,92,93^. Originally identified as an inflammasome regulator via CRISPR screening by Schmid-Burgk et al.^65^, NEK7 is an intracellular K^+^ sensor, which interacts with NLRP3 to signal for oligomerization of the latter upon K^+^ efflux from the cell^64^. NEK7 only operates outside of the mitosis portion of the cell cycle, meaning that the cells that are susceptible to inflammasome activation are limited to those in interphase^66^, and thus not all cells in a stimulated culture display NLRP3 inflammasome activity simultaneously. Elevated extracellular K^+^ levels in the range of 50–130 mM have been reported to inhibit various mechanisms of NLRP3 inflammasome activation, both canonical and noncanonical^91,94^ (Pike et al., in press), by eliminating the electrochemical gradient that drives K^+^ efflux leading to NEK7 signaling.

While not unique to inflammasome activation^93^, K^+^ efflux can be induced in a number of ways to provoke inflammasome activation. One commonly employed mechanism to do so artificially is the use of the bacterial pore-forming toxin nigericin, which is a K^+^/H^+^ antiporter that transports protons into the cell, decreasing intracellular pH, in exchange for departing K^+^ ions^95^. In the absence of the introduction of a foreign channel into the plasma membrane, K^+^ efflux can also occur via endogenously expressed K^+^ channels. Many such channels exist, and these are categorized into various families and subfamilies with different means of activation^96^. The two-pore domain K^+^ channel TWIK2 has been implicated specifically in K^+^ efflux leading to NLRP3 inflammasome activation in mouse lung- and bone marrow-derived macrophages in response to ATP and LPS^97^. TWIK2, however, has been reported to be predominantly expressed in the periphery rather than in the CNS^98^, making it unlikely to participate in microglial inflammasome activation.

## Kv1.3

In contrast to TWIK2, the delayed rectifier voltage-gated K^+^ channel Kv1.3, coded for by the intronless gene KCNA3, is widely expressed in the CNS. Kv1.3 is a member of the Shaker family of K^+^ channels^99^ with four subunits, each containing six transmembrane segments and a voltage sensor that opens upon detection of membrane depolarization to allow for K^+^ efflux^100^. While it is one among many Kv1 channels, which are also expressed in dopaminergic neurons^101^, evidence increasingly points to a critical, specific role for microglial Kv1.3 in neuroinflammation, NLRP3 inflammasome activation, and neurodegeneration. Microglial expression of Kv1.3 is low at baseline in comparison to neuronal expression but increases significantly upon microglial activation with LPS in rodents^102–104^. Microglial activation with LPS results in increased K^+^ conductance that is mediated by Kv1.3^105^. The activity of microglial Kv1.3 upon LPS treatment has been associated with neuroinflammation and microglia-mediated neurotoxicity in rats, being required for neurodegeneration^104^. Microglial Kv1.3 has been found to be required for neuroinflammation in LPS-activated microglia in an in vivo mouse model, where Kv1.3 knockout prevented microglial activation and IL-1β production^106^. Kv1.3 was demonstrated to be upregulated in post-mortem brains of patients with PD as well as in the MPTP, α-syn_PFF_, and MitoPark in vivo parkinsonism models^100^. Kv1.3 was significantly upregulated both in the MMC mouse microglia cell line and in vivo in response to treatment with aggregated α-syn, and this upregulation was both linked to increased channel activity and largely reversible in the presence of the specific Kv1.3 blocker PAP-1^100^. Crucially, this Kv1.3 upregulation played a significant role in neuroinflammation-mediated neurodegeneration in the MitoPark and MPTP in vivo parkinsonism models that were ameliorated upon PAP-1 administration^100^. These observations link Kv1.3 with the major facets of PD pathology.

Since the MPTP, α-syn_PFF_, and MitoPark parkinsonism models as well as LPS treatment of microglia are all associated with both enhanced Kv1.3 expression^100,102–105^ and augmented NLRP3 inflammasome activation^14,67,83^, the question arises as to whether there may be a direct link between Kv1.3 activity and inflammasome activation. Indeed, Kv1.3 inhibition with PAP-1 was shown specifically to decrease NLRP3 expression and IL-1β production in mouse microglia to control CNS inflammation in ischemia/reperfusion models^107^, and Kv1.3 has been linked to NLRP3 inflammasome activation in T lymphocytes as well^108^. Kv1.3 K^+^ currents required caspase-8 activity in Jurkat T lymphocytes^109^, although inflammasome involvement was not directly examined in this study. PAP-1 treatment diminished NLRP3 expression in MMC mouse microglia cells activated with aggregated α-syn^100^. Moreover, preliminary work from our group suggests that Kv1.3 blockade inhibits NLRP3 inflammasome activation in primary human microglia, which we previously found to be K^+^ efflux-dependent (Pike et al., in press)^67^. While microglial production of other, non-inflammasome-related cytokines is also attenuated by Kv1.3 blockade^106^, we suggest that the K^+^ efflux aspect of NLRP3 inflammasome activation may account in part for the deleterious role of microglial Kv1.3 in PD-associated neurodegeneration and neuroinflammation.

## Microglia and PD pathology: dopamine

The state of striatal dopamine release is of considerable importance in the diagnosis and research of PD. The deficiency of dopamine signaling in this brain region due to the degeneration of dopaminergic neurons in the SNpc, which project into the striatum, is responsible for the characteristic motor symptoms observed as the disease progresses^110^. In a clinical setting, imaging assessments of striatal dopamine functionality can be used to determine the stage of dopaminergic decline, for example by ^18^F-DOPA PET^111^ or DaTscan^112^, but early detection is difficult because striatal dopamine input has typically already diminished by 70–80% by the time motor symptoms begin to manifest^29,113–116^. Dopamine release takes place in a diffusion-based, three-dimensional volume transmission manner to act over a relatively broad distance of several micrometers^117^. It is salient that while dopamine release from midbrain dopaminergic neurons occurs axonally in the striatum, it also occurs somatodendritically in a localized fashion in the substantia nigra (SN) and the ventral tegmental area (VTA)^86,117–124^. Dopamine release in the midbrain is influenced by K^+^ flux; somatodendritic dopamine release is dependent on action potentials and various K^+^ channels^120,121^, and striatal dopamine release was shown to be regulated by neuronal Kv1 channels including Kv1.3^101^.

Anti-inflammatory effects of dopamine upon its binding to DRD1 and/or DRD2 receptors on microglia have been reported^85,125^. Dopamine was shown to dampen inflammation in primary rat microglia by regulating the pro-inflammatory renin-angiotensin system through DRD1 and DRD2 signaling^125^. Dopamine also attenuated neuroinflammation in both primary mouse microglia and in the MPTP parkinsonism model in vivo, specifically by blocking NLRP3 inflammasome activation and IL-1β secretion induced by various canonical stimuli through DRD1-mediated signaling for the E3 ligase-mediated ubiquitination and autophagic degradation of NLRP3 protein^85^. The SNpc and striatal microglia of both PD and normal control patients express dopamine receptors DRD1-DRD4 (but not DRD5) in situ, as do primary human microglia cultured in vitro after isolation^47^. Previous work by our group documented the ability of dopamine to block not only canonical and noncanonical but also α-syn-induced NLRP3 inflammasome-mediated IL-1β secretion from isolated primary human microglia, and DRD1 agonism in both primary human microglia and in vivo in the SYN120 transgenic parkinsonism model decreased microglial inflammasome activation (Pike et al., in press). As a whole, these observations point to an inhibitory role for dopamine on PD-relevant microglial NLRP3 inflammasome activation and microglia-mediated neuroinflammation.

In an in vitro coculture model with neuronal cells, isolated primary human microglia showed a tendency that was augmented upon activation of the microglia with LPS to cluster preferentially around tyrosine hydroxylase-expressing neuronal cells as compared to non-dopaminergic phenotypes^47^. The LPS-activated microglia also engaged in a strong chemotactic response to dopamine, and the authors suggested that the effects of dopamine on activated human microglia may explain the selective vulnerability of dopaminergic neurons to microglia-mediated degradation in PD^47^. These authors interpreted their findings as an indication of a role for dopamine in the facilitation of dopaminergic neurodegeneration by activated microglia. However, another interpretation is possible in light of the more recently observed effects of dopamine on inflammasome activation: that the selective attraction of α-syn- or otherwise-activated microglia to dopamine-producing neurons could be accounted for by the ability of dopamine to inhibit the inflammasome as a means of maintaining homeostasis by mitigating neuroinflammation.

## Discussion: hypothesis for the convergence of NLRP3 inflammasome, potassium, and dopamine mechanisms in PD pathology

Each of the three key characteristics of PD, namely α-syn pathology, neuroinflammation, and dopaminergic neurodegeneration, has been linked independently to the NLRP3 inflammasome as well as to Kv1.3 channel activity. Mechanistic connections between the NLRP3 inflammasome and Kv1.3 are becoming increasingly clear, and we suggest that K^+^ efflux leading to microglial NLRP3 inflammasome activation associated with PD pathology may proceed through Kv1.3. Dopamine release from dopaminergic neurons is influenced by K^+^ flux, and dopamine has been shown to be able to block NLRP3 inflammasome activation in primary human and mouse microglia.

**Fig. 1:**
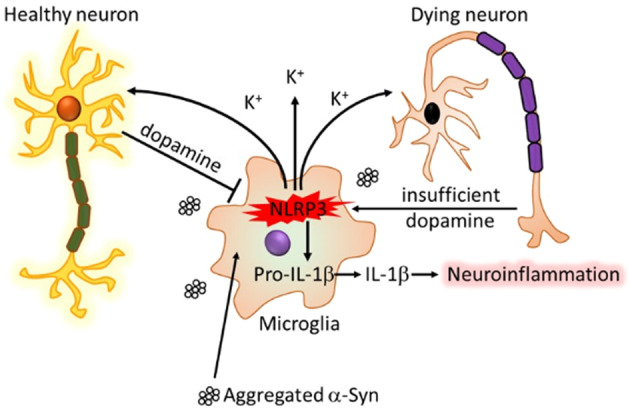
Proposed convergence of NLRP3 inflammasome, K^+^ flux, and dopamine mechanisms to elicit PD pathology. α-Syn binds to surface receptors and is taken up by microglia, leading to Kv1.3 (possibly assisted by other K^+^ channels) -mediated K^+^ efflux signaling for the activation of the NLRP3 inflammasome and caspase-1. The efflux of K^+^ could provoke K^+^ channel-mediated or toxicity-induced release of dopamine from proximal dopaminergic neurons. Dopamine could then bind to its receptors on microglia to curb activation of the inflammasome. Sufficient DA in the system would keep this interaction under control, but if DA is depleted over time by the microglia, the imbalance could allow for the progression of inflammasome-associated α-syn aggregation, neuroinflammation, and neurotoxicity, and thus PD pathology.

We propose a hypothesis for PD pathology (first described in Pike et al., in press and illustrated in Fig. 1) in which sustained activation of microglia in the SNpc by α-syn or other known stimuli induces K^+^ efflux through Kv1.3, with or without the help of other channels, to initiate NLRP3 inflammasome activation with resultant IL-1β secretion. The IL-1β would potentiate neuroinflammation while the K^+^ efflux from the microglia could present localized fluctuations in extracellular K^+^ concentrations, provoking somatodendritic dopamine release from proximal dopaminergic neurons. (Alternatively, dopamine release may occur nonspecifically as microglia-mediated neurodegeneration, fueled as previously demonstrated by both the NLRP3 inflammasome and microglial Kv1.3 activity, progresses.) Released dopamine could then bind to its receptors on adjacent microglial surfaces. Each of these processes could be enhanced by dopamine-mediated chemotactic recruitment of additional inflammasome-activated microglia. The dopamine would then be able to provide a negative feedback signal for inflammasome activation and neuroinflammation, but gradually deplete the neuronal dopamine supply in the process. Functional dopamine signaling in the SNpc and striatum could be preserved in the presence of ample dopamine but would become increasingly impaired as neuronal stores are exhausted by microglial demand. If the dopamine countersignal thus fades, the extent of microglial inflammasome activation could overtake that of dopamine-mediated inhibition, escaping regulation to a point of imbalance and a net amplification of NLRP3 inflammasome- and Kv1.3-associated PD pathology, with the dopaminergic neurons becoming overwhelmed and succumbing to the microglia. In this context, being equipped with a dopamine supply would be protective for the neurons, but being a dopaminergic neuron would be a liability if the dopamine supply is or becomes inadequate.

If a mechanism such as that which we propose should prove to participate in PD pathology, it could help to explain the largely selective vulnerability of dopaminergic neurons in PD, and particularly those in the SNpc in comparison to those in the VTA or other brain regions^87,114^. Dopaminergic neurons are characterized by their expression levels of tyrosine hydroxylase (TH), which is the rate-limiting enzyme in the synthesis of dopamine^114,126^. The SNpc and VTA areas of the midbrain are dopamine-rich, containing the two largest populations of TH^+^ neurons out of nine dopaminergic groups in the mammalian brain^114^. The substantial density of microglia in the SN, the highest in the brain^24,46,48^ at 12% of its cell population^127^, is threefold that of the VTA^128^, and this likely renders its neurons more susceptible to microglia-mediated attack than neurons in other locations including the VTA once the microglia are activated. There are other brain regions that also contain a relatively dense population of microglia, like the hippocampus;^127^ however, neurons in these regions lack their own dopamine stores, instead of receiving dopamine input from the VTA and the locus coeruleus (LC)^129,130^. Dopaminergic neurodegeneration in the VTA and LC can be observed in PD, but neuronal loss occurs to a lesser extent in these regions than in the SNpc and is not specific to PD, being apparent in other diseases like Alzheimer’s (AD) as well^87,114^. AD patients display upregulated Kv1.3 in the frontal cortex^131^ as well as increased caspase-1 activation in the frontal cortex and hippocampus, suggesting increased NLRP3 inflammasome activation^132^. However, without a dopamine supply comparable to that of the SNpc, there is less dopamine available there to play inhibitory and chemotactic roles in the context of the hypothesis we propose. We suggest that the interplay between Kv1.3, dopamine, and inflammasome activation that we propose takes place to drive pathology to whatever extent these three factors are present and functional in a given brain region, and thus can vary in intensity by location.

In addition to their microglia population and their ready dopamine supply, the dopaminergic neurons of the SNpc are postulated to have axons that are long, highly branched, and poorly myelinated^87,133,134^. The extensive branching places a high metabolic demand on SNpc dopaminergic neurons in comparison to neurons in other areas^87^. Insufficient insulation by myelin could confer vulnerability to small, local fluctuations in K^+^ flux and electrical signaling that might be largely avoided in other, better-insulated areas. While spatial buffering of K^+^ by local astrocytes would theoretically help to avoid this issue, astrocytes of the ventral midbrain, including in the SN, have low membrane resistance and low baseline levels of K_ir_-mediated currents relative to astrocytes of the hippocampus or cortex^135^. This finding has been suggested to indicate a comparatively diminished K^+^ buffering function of astrocytes in the ventral midbrain that could have an electrical impact on the regulation of burst firing of the dopaminergic neurons they serve^135^. If this is the case, these neurons might be rendered more susceptible than those in other regions of the brain to the stress of superfluous extracellular K^+^ generated locally by microglial Kv1.3 activity.

In summary, we suggest three main factors for brain region susceptibility to PD pathology, mediated by the NLRP3 inflammasome, K^+^, and dopamine mechanism-based interaction that we propose in our hypothesis, which overlaps in the SNpc to a greater extent than in the VTA or other brain regions: (1) high microglia density with attendant NLRP3 inflammasome expression and activity, (2) a ready dopamine supply, stored in dopaminergic neurons, and (3) poor myelination of these neurons, leaving them more vulnerable than other neuronal populations to swings in ion flux and electrical signaling. Variability across brain regions in transcriptional profiles of microglia^46^ and differential distribution of α-syn expression and isoforms/conformations are also very important factors in PD development. However, examining these intact from the human brain proves very difficult because microglia are so plastic and their transcriptional profiles so responsive to their surroundings^136^ and because much of the conformational structure of α-syn is lost upon water dissipation after death and upon treatment with fixatives such as paraffin, which would disrupt lipid-soluble conformations^137^. While α-syn pathology undoubtedly plays an important role in PD progression and is associated mechanistically with inflammasome and caspase-1 activities^14,37^ as well as microglia-mediated dopaminergic neurodegeneration^21^, Lewy body/Lewy neurite pathology per se does not always correlate with neurodegeneration or loss of motor function^87^; moreover, it is not unique to PD, as other synucleinopathies like DLB and MSA also display α-syn pathology but in brain regions other than the SNpc^45,138^.

## Conclusions

In this review, we provide the framework for a hypothesis in which microglial NLRP3 inflammasome, K^+^ flux, and dopamine response mechanisms interact to maintain homeostasis between microglia and neurons by modulating microglial activation through dopamine-mediated negative feedback on the inflammasome. If this synergy is disrupted such that the dopamine supply is depleted and NLRP3 inflammasome activation intensifies, the three major facets of PD pathology (α-syn accumulation, neuroinflammation, and dopaminergic neurodegeneration) would be augmented as a result of weakened microglial regulation. We propose that high microglia density, local dopamine stores, and inadequate axonal myelination serve as key determinants that confer risk for pathology mediated by the mechanism that we put forth, and that these factors overlap to a greater extent in the SNpc than in other brain regions like the VTA or LC, where the confluence would be less, helping to explain the predominant targeting of this area in PD. While the literature evidence provided here lays the groundwork for this idea, more work remains to be done to provide further support. Experiments designed to explore the relationships between the inflammasome, its specific mode of K^+^ efflux, and the effects of dopamine on these must be performed.

If a mechanism such as that which we propose is operational in PD pathology, it could shed more light on the etiology of the disease, and furthermore, open the door to treatment options alternative to or in conjunction with L-DOPA therapy to potentially lessen the need for long-term l-DOPA administration with its ancillary risks. While l-DOPA treatment has long been the gold standard for PD therapy^139,140^, its long-term use is associated with the risk of l-DOPA-induced dyskinesia^141^. For this reason, l-DOPA treatment for most patients does not occur until long after symptoms have become evident enough to lead to a PD diagnosis. By the time motor symptoms begin to manifest, as mentioned above, striatal dopamine input has typically already diminished by 70–80%^29,114,115^. Even if l-DOPA administration were begun immediately upon diagnosis, the process we describe in our hypothesis likely would have been happening for a very long time prior. Because of the extent of neurodegeneration at that point, other factors outside of those involved in the hypothesized mechanism would be contributing to further degradation of neurons in the area (e.g., reactive oxygen species, ATP released from dying neurons, etc.), and l-DOPA administration would likely not be able to halt these more general mechanisms. Thus, the key to stopping the pathological feedback mechanism we propose would lie not in late-stage dopamine replacement therapy, but in preventing the initial loss of dopamine leading to neurodegeneration by controlling the activities of Kv1.3 and the NLRP3 inflammasome.

Sarkar et al.^100^ showed initial evidence that Kv1.3 is not only elevated in microglia but also in peripheral B cell lymphocytes from both early- and late-stage PD patients in comparison to those from healthy controls. This led them to the suggestion that Kv1.3 expression in peripheral lymphocytes could serve as a potential biomarker for PD, though this remains to be confirmed with studies involving larger sample sizes. If this proves to be the case, we suggest that early screening for prodromal PD could involve an initial plasma evaluation for elevated B cell lymphocyte Kv1.3 expression which could be followed up with ^18^F-DOPA PET imaging to examine dopamine signaling functionality. If both of these tests are abnormal, and cancer is ruled out through analysis of the original blood sample or other appropriate methods (as Kv1.3 expression can be associated with some cancers^142^ and abnormal ^18^F-DOPA PET results can be indicative of neuroendocrine tumors^143^), the combination could be indicative of prodromal PD and intervention could be possible prior to large scale neurodegeneration and the development of motor symptoms. NLRP3 inflammasome inhibitors are already being tested for their efficacy in slowing/preventing PD pathology, as are Kv1.3 blockers. If such treatment strategies are effective and could be administered prior to significant neurodegeneration, they could prove to be truly disease-modifying therapies for PD.

Moreover, a recent genome-wide association study (GWAS) identified 90 genetic risk factors for PD^144^, several of which may be pertinent to the mechanism we hypothesize. Those of particular interest in this context include the *FYN* gene encoding Fyn kinase, which has been shown to regulate microglial α-syn uptake and NLRP3 inflammasome activation^145^ in addition to modulating Kv1.3 expression^100^. Among many other genes with low penetrance relative to previously identified causative, higher-penetrance PD genes such as *SNCA*, *PARK2*, and *LRRK2*, this GWAS study also identified as PD risk factors *NFKB2*, encoding a subunit of the Nf-κB complex, *SCAF11*, encoding a caspase, and *KCNS3* and *KCNIP3*, both encoding voltage-gated potassium channel subunits (Kv9.3 and calsenilin). While the *NLRP3* and *KCNA3* genes, encoding NLRP3 and Kv1.3, respectively, were not identified specifically in the study, it is possible that each as an independent genetic risk factor may be neutralized by compensatory mechanisms. However, if *FYN* upstream is affected such that both NLRP3 and Kv1.3 signaling are enhanced simultaneously, particularly together with Nf-κB, given its known role in upregulating NLRP3 and Kv1.3 expression, and other potassium channels, these genes if present in some combination may form a polygenic risk panel for PD pathology proceeding according to our hypothesis that could be identified upon genetic screening.

## Data Availability

The data supporting this study are available from the corresponding author upon reasonable request.
